# *Arthrospira platensis* as Protein-Rich Source for Human Nutrition

**DOI:** 10.3390/life15121789

**Published:** 2025-11-21

**Authors:** Steffen Braune, Conrad G. H. Jung, Jan-Heiner Küpper, Friedrich Jung

**Affiliations:** 1 Institute of Biotechnology, Molecular Cell Biology, Brandenburg University of Technology Cottbus-Senftenberg, 01968 Senftenberg, Germany; jan-heiner.kuepper@b-tu.de (J.-H.K.); friedrich.jung@b-tu.de (F.J.); 2 Faculty of Health Sciences Brandenburg, Brandenburg University of Technology Cottbus-Senftenberg, 01968 Senftenberg, Germany; 3 Carbon Biotech Social Enterprise AG, 01968 Senftenberg, Germany

**Keywords:** *Arthrospira platensis*, *Limnospira platensis*, protein quality, protein quantity

## Abstract

The continuing growth of the world’s population, combined with climate change, poses a growing challenge to ensuring food security in the 21st century. Animal protein, e.g., from beef, is a particularly rich source of protein, but there is by no means enough arable land on earth to transfer the animal meat-rich nutritional style of the early industrialized countries to the global South. A hitherto largely neglected option for the production of proteins is the cultivation of microalgae and cyanobacteria, which already have a long history of use as a human or animal food for their nutritional and environmental merits. In particular, *Limnospira platensis* (Spirulina and formerly *Arthrospira platensis*)—a filamentous cyanobacterium—is considered the “food of the future” since it is a viable source of vegan protein. In this manuscript, we review the scientific literature as well as national and intergovernmental agency statements regarding the quality and quantity of AP-based proteins. The content of AP protein is discussed in relation to other species and different cultivation conditions, as well as to traditional crops and animal husbandry. The amino acid profile and quality assessment of AP as a dietary protein are discussed. In addition, the environmental aspects of AP production are considered, and the role of AP in efforts to bridge the ‘protein gap’ to improve nutrition and food security is discussed.

## 1. Introduction

The world’s population continues to grow and will reach 9.7 billion people by 2050, according to United Nations projections [1]. Along with the increasing use of natural resources, climate change, and biodiversity loss, this poses a growing challenge to ensuring food security in the 21st century. To maintain energy metabolism, humans need sufficient calories in the form of carbohydrates and fats. In addition, a continuous supply of essential amino acids, vitamins, and minerals to maintain basic cell functions is needed. Malnutrition is usually a combination of insufficient intake of calorie-rich foods and, in particular, essential amino acids for protein anabolism [2].

Protein is the major structural component of all cells in the body. Proteins also function as enzymes, as transporters or receptors in membranes, as transport carriers of, e.g., lipids, and as hormones. Amino acids, the building blocks of proteins, serve as precursors for the diversity of cellular proteins to function in immune reactions, muscle contraction, and action potentials, and they are an important nitrogen source for other cellular molecules. The “Recommended Dietary Allowance” for both men and women is 0.8 g of good-quality protein/kg body weight per day [3]. The most important aspect of a protein from a nutritional point of view is its amino acid composition, but the protein’s structure may also influence its digestibility.

Animal protein from beef, pig, or chicken, for example, is a particularly rich source of the nine essential amino acids. But there is by no means enough arable land on earth to transfer the animal meat-rich nutritional style of the early industrialized countries to the global South or developing countries in principle [4,5]. Furthermore, a diet based on animal products leads to a disproportionate amount of CO_2_ emissions. The Intergovernmental Panel on Climate Change (IPCC) estimates that food production and distribution together are responsible for 21 to 37% of all man-made greenhouse gases [6]. New nutritional concepts are needed to ensure the long-term food security of the world’s population and, at the same time, to preserve our planet.

A hitherto largely neglected option for the large-scale production of proteins for human and animal food [7,8,9,10,11,12,13] as a renewable raw material is the cultivation of microalgae and cyanobacteria [14,15]. However, as early as 1974, the cyanobacterium *Limnospira platensis* (LP, often referred to as Spirulina [16] and earlier as *Arthrospira platensis* [17,18]) was identified as the “food of the future” at the United Nations World Food Conference.

**Figure 1:**
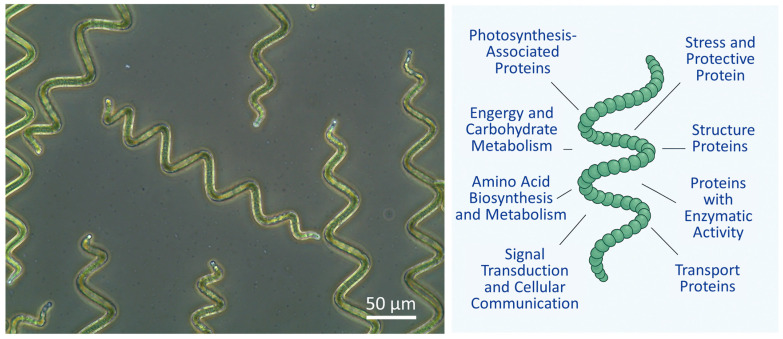
(**Left**) Typical morphology of spirals and (**Right**) overview of functional categories of *Arthrospira platensis* proteins (Left: phase contrast microscopy, 40-fold primary magnification, EVOS™ XL Core Imaging System, Thermo Fisher Scientific, Darmstadt, Germany).

This filamentous, helical-shaped, photoautotrophic, and alkaliphilic *Limnospira platensis* contains over 50 nutrients and is rich in proteins containing all essential amino acids. Figure 1 representatively shows the typical morphology of AP cultured in Zarrouk medium, as well as the major functional categories of proteins in AP [19].

It is further rich in B vitamins, iron, magnesium, potassium, and many other vitamins and minerals, as well as antioxidants [17,20]. AP has received considerable attention due to its production process in open pond systems or closed bioreactors, which requires less water and land than crops or animal-derived foods [21]. Due to these characteristics, AP is currently the most exploited industrial species, aside from *Chlorella* (Chlorophyta), *Dunaliella*, *Crypthecodinium* (Dinoflagellata), *Haematococcus* (*Chlorophyta*), and others [22,23,24].

In this brief review, we discuss the characteristics of AP protein with regard to its intracellular quantity and nutritional quality.

## 2. Protein Quantity

Many microalgae and cyanobacteria species contain high levels of protein, with AP typically comprising 40–60% protein of dry matter (see Table 1 [25,26]). This is one of the highest protein contents of microalgae species and of food in general [19,27,28], and it is one of the reasons why they have historically been used as a human food source [29]. As a bacterium, AP has the advantage over other photoautotrophic microorganisms in that it does not have a cell wall made of cellulose; therefore, it does not require chemical or physical processing to be metabolized [25].

Depending on the strain and the environmental and production conditions, the protein content can range between 17% and 80% of the dry weight (Table 1). Cultivation conditions that affect the protein content include the composition of the culture medium, the habitat, the light source and intensity, the temperature, and the method for the protein extraction and analysis, as well as the growth phase in which the biomass was harvested [37,38]. One of the main influencing factors is the illumination [39,40,41]. Light color (spectra), light intensity, and duration of illumination (light/dark cycles) can influence the quantity and quality of the biomass gained in AP cultures significantly [42].

The protein content in AP is higher than in plant or animal sources such as wheat, maize, rice, soybeans, or dried beef (55.6% [43,44,45] (Table 2). Under well-standardized conditions, protein concentrations of around 60% can be achieved in different cultivation setups (Table 3). Lower values are associated with more experimental approaches, which typically are aimed at optimizing culture conditions or understanding the influence of individual cultivation parameters (e.g., pH, temperature, CO_2_, etc.) on growth and AP biomass composition [36,46,47]. The same applies to studies focusing on different nutrient sources/compositions for economic or environmental reasons, e.g., carbon or nitrogen from residues of other production processes [48]. Also, approaches for the recycling of used culture media were evaluated in this matter [49,50]. The results shown in Table 2 summarize that about 40 times more protein can be produced per hectare than with plant crops and also significantly more than with meat production.

## 3. Protein Quality

Not only the quantity but also the quality of the protein plays a major role. From a qualitative point of view, AP proteins are complete, since all nine essential amino acids present meet the Food and Agriculture Organization of the United Nations (FAO) requirements. It has been demonstrated that AP protein is a source of essential amino acids that is comparable to other proteins, including those derived from soybeans and chicken eggs [59,60]. The free amino acids dissolved in the body fluids are only a very small proportion of the body’s total mass of amino acids. Nevertheless, they are very important for the nutritional and metabolic control of the body’s proteins.

### 3.1. Amino Acid Profile

Since the amino acid profile of dietary proteins is not identical to the amino acid profile of body proteins, proteins are considered to be of lower quality the further their amino acid profile deviates from the body’s own amino acid profile. Therefore, it is not the number and quantity of amino acids in dietary protein that limit protein synthesis, but rather the concentration of the amino acid that is most deficient (called the limiting amino acid). If a limiting amino acid is depleted, the body cannot produce endogenous proteins, since humans can only convert amino acids into endogenous protein to the extent that the smallest amount of an amino acid is available. As with the biological value, the chicken egg white serves as a reference value of 100 (or 1.0). As shown in Table 4, the spectrum of amino acids of AP shows that the biological value of proteins in spirulina is comparable to egg white [60,61,62].

Histidine and lysine were determined to be limiting or slightly under-represented in the case of AP, while in another study, lysine concentration was reported to be adequate [61]. This study revealed that the amino acids methionine and cysteine were the limiting amino acids. Even so, they are present at more than 80% of the ideal level defined by the FAO, calculated on the basis of egg albumin and casein.

It must be taken into account that the drying methods—especially drying on hot drums—can reduce the methionine content by some 30% compared to spray drying by evaporation [66,67,68].

### 3.2. Quality Scoring of Dietary Protein

In addition to the amino acid composition, different scorings were developed to assess the quality of dietary protein. The Protein Digestibility Corrected Amino Acid Score (PDCAAS, recommended by the FAO, became the industry standard in 1993 [69]. FAO has recently recommended the newer Digestible Indispensable Amino Acid Score (DIAAS) to supersede PDCAAS. The DIAAS system (ileal digestibility of the essential amino acids) was emphasized as a more accurate measure of protein absorption [70,71,72]. The difference between PDCAAS and DIAAS lies in the fact that fecal digestibility, used in PDCAAS, may be affected by microbial degradation, while true ileal digestibility, used in DIAAS, more accurately represents the amounts of amino acids absorbed in the gastrointestinal tract [68,72]. For this reason, DIAAS is promoted as the superior method and preferred over the PDCAAS [73,74].

Devi et al. reported that the DIAAS of AP is 85.2% higher than DIAAS values of certain plants (chickpeas: 56.6% or mung beans: 57.7%) [73]. Burd et al. showed that raw beef meat had clearly higher values (97%) than all plant or microalgae DIAAS [74]. However, grilled or baked (80%) beef meat showed lower values than raw beef meat or even AP.

In other countries (e.g., Canada), the Protein Efficiency Rate (PER) is additionally the official method for assessing the quality of protein [75,76]. This is the weight gain of an individual divided by the weight of proteins ingested. Measurements are usually made on growing rats. The reference proteins are lactalbumin or casein [77].

The PER value for AP determined in growing rats was estimated between 1.8 and 2.6 [78,79,80], as against a PER value for casein of 2.5. The PER values of plants are mostly lower, as for maize (1.23) or wheat (1.15) [78].

### 3.3. In Vivo Studies

The growth rate of rats fed AP as the sole protein source is higher than or equal to that of control animals. In addition, rats fed AP fixed greater or equal amounts of protein compared to rats supplemented with essential amino acids of equal metabolic energy. These results indicate excellent metabolic use of the amino acids in AP, which is confirmed by the levels of free amino acids found in the blood and muscle of test animals [73].

There are a few studies performed on humans. These studies tend to show results similar to those obtained in animal trials, though digestibility seems slightly lower [61,81,82].

## 4. Technological Challenges in Production and Processing

### 4.1. Cultivation Strategies

Cultivation and processing of microalgae require complex processes. Although AP and other microalgae species are characterized by relatively high growth rates and photosynthetic efficacies, upscaling to an industrial level and the associated cost structure are still challenging [83,84].

Autotrophic cultivation—e.g., in open raceway ponds—is relatively inexpensive and still represents the standard for the large-scale production of AP. However, it is reported to yield lower biomass densities in comparison to, e.g., mixotrophic approaches [85,86]. The harvesting and the energy expenditure are the dominant cost factors for autotrophic cultivation systems [87].

In principle, heterotrophic cultivation can result in high biomass and protein yields, but it is more cost-intensive [86,88]. Associated with this are higher production costs, e.g., for sterility of cultivation and processing systems, as well as a strong dependency and sensitivity of the organisms to the cultivation medium and chemical substrates (e.g., glucose) [85,86,89]. Until now, attempts to identify AP strains capable of continuously growing under dark heterotrophic culture conditions have not been successful. Thus, exposure to light plays a crucial role in reaching an industrial scale cultivation and respective growth rates [89,90].

In mixotrophic systems, high growth rates can be achieved by combining the two aforementioned cultivation techniques. This approach requires strong monitoring and regulation of factors such as the carbon source and lighting regime, which increases the complexity of the overall process [85,91,92]. Pereira et al., [91] for instance, presented a successful mixotrophic cultivation approach using dairy wastewater. In this study, an increase in the biomass concentration during 15 cultivation days was achieved by supplementing the reference Zarrouk medium with 5% buffalo mozzarella cheese whey. However, under these cultivation conditions, the protein content of the AP biomass decreased while the carbohydrate content increased [91].

### 4.2. Biotic and Abiotic Contamination

A major challenge associated with the cultivation of AP for the production of food/feed-related products—and thus the biomass quality and safety—is the management of biotic and abiotic contaminants.

In this context, contamination with other organisms such as toxic and non-toxic heterotrophic bacteria, insects (e.g., Ephydridae), and harmful species of micro-zooplankton (flagellates, amoebae, ciliates, and rotifers) is reported, which can occur in high densities and graze AP [93,94,95].

Particularly in open cultivation systems such as ponds, contaminants can be introduced by animals or airflow, which can survive in the culture despite the relatively high pH value. Contaminations through human handling during the processing of the culture, but particularly of the biomass, are very likely less relevant, but are discussed as well [96].

Vardaka et al. tested 31 commercial AP food supplements and identified 469 different taxonomic units [97]. Pathogenic bacteria—such as *Pseudomonas*, *Clostridium*, and *Enterococcus*—represent major challenges, posing health risks to humans and animals [98]. Likewise, cyanobacterial species that produce toxins such as microcystins (e.g., *Microcystis*) can be a serious source of contamination in the production of food/feed-related products from AP biomass [99,100].

Beyond biological contaminations, AP cultures can be affected by abiotic factors such as heavy metals and pesticides [101] since the organism can absorb these substances from its environment (biosorption). On the one hand, this is studied as a potential remediation technology (phytoremediation), and on the other, it acts as a source of contamination [93]. Particularly in areas with high industrial pollution and when low-grade agricultural fertilizers are applied as a nutrient source, even relatively low amounts can accumulate in the AP biomass with cultivation time. The same applies for cultures that come into contact with contaminated soil, e.g., in earthened ponds or when heavy metal-contaminated lining materials are used for ponds [102].

Successful production requires a comprehensive understanding of the various sources of contamination (biotic and abiotic) and the implementation of effective prevention and control measures [15]. While open pond systems are more cost-effective and accessible, closed cultivation systems combined with modern sterilization and monitoring technologies provide a promising way to produce high-quality AP biomass free from contamination [103,104,105].

### 4.3. Functional and Sensory Characteristics

The functional properties of AP protein biomass, such as foaming, gelling, or emulsifying ability, are crucial for the further processing of AP but depend significantly on environmental factors such as pH value, ion concentration, and temperature [106,107,108]. A high foaming capability was observed for AP protein isolates, but with low stability in many pH ranges. Gel formation is also possible, but more difficult at acidic pH and less efficient than with established protein sources such as soy.

In terms of sensory properties, taste, color, and smell represent challenges for consumer acceptance and require technological solutions for masking or processing [109]. For instance, phycocyanin colors protein isolates substantially, and bioactive peptides, as well as free amino acids in the biomass, are associated with bitter taste [110,111].

Lipid oxidation during storage and drying procedures can result in the production of volatile compounds (so-called off-flavors) that can lead to ‘fishy’ or ‘seaweed-like’ odors [112,113]. Sensory evaluation studies show that increased proportions of AP in a food product can limit acceptance due to these sensory properties. Accepted levels of added AP biomass vary among the studied products and range, e.g., for pasta and nectar, between 10 and 20% [114,115] and 1 and 2% for fruit cereal bars, yogurt/cheese products (labneh), vegan pesto, and a sourdough bakery product [116,117,118,119]. Thus, further technological solutions for masking undesired sensory properties and processing are required for improving consumer acceptance.

## 5. Environmental Considerations

The production of AP proteins offers environmental advantages over conventional protein sources. This approach to protein production is more resource-efficient, requiring less water and land compared to traditional sources like soy or cattle farming. AP can be grown on barren land, making use of areas not suitable for conventional food production.

From an environmental standpoint, AP biomass production stands out for its sustainability. The process does not involve genetic manipulation, pesticides, or herbicides. The alkaline pH environment in the cultivation medium can reduce undesired organisms (co-flora), reducing the need for chemical intervention [120,121]. Essential nutrients can be efficiently incorporated and monitored without the risk of over-fertilization. This nutrient management prevents groundwater contamination, a significant advantage over conventional farming methods. In arid regions, the ability to use sea or brackish water for production further contributes to the conservation of precious groundwater resources [122].

AP production can contribute to climate protection because AP, like other microalgae and cyanobacteria, outperforms many photosynthetic plants in carbon dioxide fixation per unit of biomass. This is due to the significant investments plants make in non-photosynthetic structures, such as roots, stems, and supportive tissues, as well as their lower overall conversion efficiencies [123,124,125,126,127]. Thus, AP-based protein production can help mitigate the emission of greenhouse gases like carbon dioxide and methane, which are typically associated with animal husbandry.

A further advantage of AP protein production lies in its potential impact on land use and reforestation. By partially or completely replacing animal proteins with AP-based products, vast areas currently dedicated to animal feed or pasture could be freed up. These liberated lands could be repurposed for planting new forests, which would serve as additional carbon sinks that actively sequester atmospheric carbon. Furthermore, the trees grown on these reclaimed areas could eventually be harvested for use as construction timber, providing a long-term carbon storage solution.

Life cycle assessment studies indicate that optimizing production and harvesting processes is important for achieving an advantage in terms of the economic and environmental footprint/costs per kg of microalgae-based protein compared to conventional protein sources [86]. This is particularly discussed for regions with relatively low levels of sunshine and average temperatures, such as Central Europe, due to the higher energy consumption/costs for the cultivation and processing of biomass.

The reduction of, e.g., freshwater requirements, utilization of waste heat, and agricultural waste streams has the potential to substantially improve the economic and environmental footprint of microalgae production [128,129].

## 6. Regulatory Aspects

From a regulatory perspective, AP is generally regarded as safe (GRAS) by the European Food Safety Authority (EFSA) [130] and by the U.S. Food and Drug Administration (FDA). The latter accepted a GRAS notification (GRN No. 417) for the use of dried AP biomass as an ingredient in various foods and drinks without objection in 2012 [131]. Thus, these microalgae have the potential to serve as a substitute for vegetable protein in food products, and their efficacy may even surpass that of soy.

One of the major challenges in the production of AP-derived protein for food and feed applications is the dependency of protein and nutrient composition on environmental conditions and cultivation location. This complicates standardization for the food industry. As described in a previous paragraph, AP can accumulate heavy metals, iodine, or toxic metabolites, which necessitates additional purification and control steps (see Section 4.2).

Despite its GRAS status, the allergenic potential has not been conclusively clarified, as discussed by Gromek et al. [132]. Strong allergic reactions are very rare and mainly reported for predisposed, atopic individuals [133,134]. The French Agency for Food, Environmental and Occupational Health & Safety reported on the “risks associated with the consumption of food supplements containing spirulina” in 2017 [135]. An expert committee concluded that “Apart from the risk of contamination, spirulina does not seem to present a health risk at low doses (up to several grams per day)”. As described above, contamination of cultivation systems with other bacteria (e.g., cyanotoxin-producing species) and heavy metals is possible and can exceed the limits laid by the regulatory authorities. These parameters must be taken into account in the discussion about and the testing of the allergenic potential of AP. The current research suggests that AP biomass has an allergenic potential with minimal clinical relevance in healthy individuals [132,136,137]. It should be noted that beyond fundamental animal trials from the 1990s (see: [132]), recent in vitro and in vivo studies report on the anti-allergic potential of AP, e.g., for the treatment of allergic rhinitis [138,139,140]. These were, however, critically reviewed, and more randomized clinical trials in accordance with the CONSORT standardization have been suggested [141].

According to the regulatory situation, the application of AP protein as a food product is uncritical due to its legal acceptance in the US, the EU, and Asia. However, this applies to AP biomass that has not undergone any significant changes in processing or composition. New technologies—such as protein isolates—or use in novel foods may require a complex approval procedure, e.g., according to the “Novel food” regulation in the EU [142].

## 7. Conclusions

Arthrospira is, with up to 80% dry mass, very rich in protein. The protein content is higher in comparison to unicellular algae and most other cyanobacteria or to beef, pig, or chicken meat. Without requiring large areas of arable land and less water compared to conventional crops/livestock farming, AP thus offers the possibility to close the “protein gap”. In addition, comprehensive analyses and nutritional studies have demonstrated that AP protein is of high quality and as good as animal proteins and even better than conventional Leguminosae-based protein.

However, the quality of proteins can vary dramatically, depending on digestibility and the availability of essential amino acids [143]. In contrast to vegetable proteins—which often contain only a small amount of one or more EAAs or lack them completely—AP is a considerable source of protein whose EAA composition meets FAO requirements in comparison to egg white. AP is well digestible. The thin cell wall of AP consists of peptidoglycan and polysaccharide layers within the typical organization of Gram-negative bacteria. This is in contrast to eukaryotic microalgae such as Chlorella, in which the cell wall contains an indigestible cellulose scaffold.

Despite the remaining technological challenges, a shift towards AP-based protein production represents a holistic approach to addressing multiple environmental challenges simultaneously. It can offer a sustainable, efficient, and environmentally friendly alternative to traditional protein sources, with far-reaching benefits for resource management, climate protection, and land use optimization.

## Figures and Tables

**Table 1:** Protein content of *Arthrospira platensis* of different strains.

Species	Protein Content [% Dry Matter]	Reference
*Arthrospira fusiformis*	35–64	[30,31]
*Arthrospira maxima*	60–71	[32,33,34]
*Arthrospira platensis*	17–80	[35,36]

**Table 2:** Total yield, protein content, and protein yield of traditional crops and cultures of *Arthrospira platensis*.

Crop	Total Yield [Tons/(ha per Year)]	Protein Content [%]	Protein Yield [Tons/(ha per Year)]
Wheat	6.7	9.5	0.64
Maize	14	7.4	1.04
Rice (hulled)	8	7.1	0.57
Soybeans	4	35	1.4
*Arthrospira platensis*	60–70	65	39–45

**Table 3:** Representative protein contents of *Arthrospira platensis* under different production conditions.

Culture System	Culture Medium	Protein Content [%]	Reference
Erlenmeyer flask	Schlösser (+Beet vinasse)	39–55 35–72	[51]
Photobioreactor	Schlösser (urea as nitrogen source)	18–71	[52]
Photobioreactor	Zarrouk (sodium nitrate modification)	56–70	[53]
Photobioreactor	Zarrouk	69	[36]
Photobioreactor	FM-II	58–63	[48]
Semicontinuous culture system	Conwey	61–64	[54]
Elongated minitank (outdoor pond simulation)	Schlösser (urea as nitrogen source)	45–62	[55]
Open pond	Kosaric and digested sago starch factory wastewater	53–68	[56]
Open pond	Modified Zarrouk	56	[57]
Open pond	n.d.	59	[58]
Erlenmeyer flask	“Standard” with variations	17–32	[35]
n.d.	n.d.	46–63	[32]

**Table 4:** Dietary amino acid requirements as stated by the Food and Agriculture Organization of the United Nations in comparison to profiles of *Arthrospira* and egg white as stated by the U.S. Department of Agriculture, Agricultural Research Service.

Essential Amino Acids (EAAs)	Required [g/100 g Protein] [63,64]	*Arthrospira* [g/100 g Protein] [61]	Egg White [g/100 g Protein] [65]
Histidine	1.6	1.08	0.257
Isoleucine	3.0	3.21	0.609
Leucine	6.1	4.95	1.02
Lysine	4.8	3.02	0.822
Methionin + Cystine	2.3	1.15 + 0.662	0.486 + 0.407
Phenylalanin + Tyrosine	4.1	2.78 + 2.58	0.726 + 0.466
Threonine	2.5	2.97	0.567
Tryptophan	0.66	0.929	0.188
Valine	4.0	3.51	0.779

## Data Availability

Data sharing is not applicable.
